# Skin Manifestations in Psoriatic and HS Patients in Treatment with Biologicals during the COVID-19 Pandemic

**DOI:** 10.3390/jcm10245841

**Published:** 2021-12-13

**Authors:** Elia Rosi, Maria Thais Fastame, Antonella Di Cesare, Gianmarco Silvi, Nicola Pimpinelli, Francesca Prignano

**Affiliations:** Department of Health Sciences, Section of Dermatology, University of Florence, 50125 Florence, Italy; elia.rosi@unifi.it (E.R.); thais9039@gmail.com (M.T.F.); antonelladicesare@yahoo.it (A.D.C.); gianmarco.silvi@unifi.it (G.S.); nicola.pimpinelli@unifi.it (N.P.)

**Keywords:** coronavirus, COVID-19, SARS-CoV-2, psoriasis, hidradenitis suppurativa, acne inversa, biologics, pandemic, skin manifestations

## Abstract

The Coronavirus Disease 2019 (COVID-19) pandemic, a global public health emergency, has changed dermatology practice and daily routine in just under two years. Much has been written in the literature about COVID-19-associated skin manifestations. Nevertheless, much less has been written regarding skin manifestations in patients affected by severe immune-mediated skin diseases, e.g., psoriasis and hidradenitis suppurativa, undergoing biological treatment during the COVID-19 outbreak. Thus, the aim of this article is to provide the reader with an overview of the cutaneous manifestations during the COVID-19 pandemic in this subset of patients.

## 1. Introduction

At the end of 2019, severe acute respiratory syndrome coronavirus 2 (SARS-CoV-2) emerged in the city of Wuhan, China and spread rapidly across the world, leading the World Health Organization (WHO) to define coronavirus disease 2019 (COVID-19) as a pandemic on 11 March 2020 [1]. Despite the fact that COVID-19-associated cutaneous manifestations have been progressively reported in recent months [2,3], to date, there is a paucity of literature exploring skin manifestations in psoriatic and hidradenitis suppurativa (HS) patients undergoing biological treatment during the COVID-19 outbreak.

Thus, in view of these considerations, the aim of this article is to provide the reader with a comprehensive schematic overview of the cutaneous manifestations throughout the COVID-19 pandemic in this subset of patients.

## 2. Methods

A literature search in the electronic database PubMed and MEDLINE was conducted up to the 23rd of November 2021, using the key terms “psoriasis” OR “hidradenitis suppurativa” in combination with “biologics” OR “biological therapy” AND “COVID-19”, to identify relevant English-language publications. Each article was selected based on its title and abstract if it involved cutaneous manifestations in this subset of patients. Further key search terms included “psoriasis flare” OR “hidradenitis suppurativa flare”, in combination with “COVID-19”. Terms “psoriasis” OR “hidradenitis suppurativa”, in combination with “COVID-19 vaccine”, was also searched. We also collected information to answer this question: could the COVID-19 pandemic have an impact on skin manifestations of psoriatic/HS patients undergoing biological therapy? References of relevant articles were also manually searched for possible inclusion in the present review.

## 3. Results

To the best of our knowledge, the present review is based upon the available literature to date.

### 3.1. Psoriasis

Psoriasis is a chronic, immune-mediated skin disease that affects an estimated 125 million people worldwide [4]. Rather recently, the National Psoriasis Foundation (NPF) COVID-19 Task Force (TF) updated the ever-evolving literature data on the effects of psoriasis treatments (including biologics) on SARS-CoV-2 infection and COVID-19 outcomes. Biological drugs, such as anti-tumor necrosis factor-alpha (TNF-α), anti-interleukin (IL)-17, and anti-IL-23 inhibitors, are widely used to manage the most severe forms [5,6]. The continuation of biologic therapy is recommended for psoriasis patients (not infected with SARS-CoV-2) in most cases [7]. Considering this recommendation, the first key question of this short review was as follows: could the COVID-19 pandemic have an impact on skin manifestations of psoriatic patients undergoing biological therapy? According to the literature, as reported below, in these patients it may be possible to observe three categories of cutaneous manifestations.

#### 3.1.1. Worsening Psoriasis

Based on case reports, psoriatic patients infected with SARS-CoV-2 may be at risk of exacerbation of their underlying psoriatic disease. In the NPF COVID-19 TF guidance for management of psoriatic disease during the pandemic, it was emphasized that psoriatic patients should be aware of this possibility, although its clinical significance is not yet known [7]. Recently, a cross-sectional survey study among 197 Egyptian dermatologists reported that 31% of respondents agreed/strongly agreed that there was an unusual exacerbation of psoriasis during the COVID-19 outbreak [8]. A total of 11 cases (listed in Table 1) reporting on psoriasis flare-up after SARS-CoV-2 infection were identified from literature search. All patients had a history of psoriasis (current or previous) but none were on biologics [9,10,11,12,13,14,15,16,17,18,19]. In addition, two cases of new-onset psoriasis (both patients had no personal history of psoriasis) secondary to COVID-19 have also been described in the literature: a 62-year-old female who developed a pustular psoriasis 4 weeks after the beginning of COVID-19 symptoms and a 25-year-old male who presented a de novo guttate psoriasis 5 days after the diagnosis of COVID-19 [20,21]. On the other hand, Mroz and colleagues observed the influence of SARS-CoV-2 infection on the course of psoriasis in biologically treated patients. In their one-year observational study, 57 patients received biologic treatment (study group) and 68 were treated with a different treatment (control group). The real-time polymerase chain reaction (RT-PCR) test for SARS-CoV-2 was positive in 8 (14%) and in 11 (16%) of psoriatic patients in the study and in control groups, respectively (*p* > 0.05). In both groups, hospitalization due to COVID-19 was not required. In the study group, six (75%; three on ustekinumab, two on adalimumab, and one on risankizumab) out of eight patients infected with SARS-CoV-2 reported a worsening psoriasis (time of exacerbation ranged from 12 to 36 days). In the control group, eight (72%; three on cyclosporin, three on Psoralen Ultra-Violet A (PUVA), and two on methotrexate therapy) out of 11 patients with COVID-19 reported a psoriasis flare-up (time of exacerbation ranged from 24 to 60 days). In both groups, ongoing therapy was not suspended [22]. A study examining treatment continuity and psoriasis course in patients treated with biological agents during the early COVID-19 pandemic was conducted by a group of researchers from Ankara, Turkey. Yalici-Armagan and colleagues reported that 28.3% (30/106) of their psoriatic patients interrupted the biological treatment voluntarily. Of these 30 patients, 66.6% (20/30) had a psoriasis exacerbation. The researchers found that the psoriasis exacerbation rate was significantly lower in patients who continued their biological therapy than those who interrupted treatment (*p* < 0.0001). None of the patients developed COVID-19 during the study [23]. Notwithstanding the outdoor activity restriction, perceived stress, and income loss, a slight worsening of psoriasis was described in only 10/180 patients on biological therapy by Filippi and colleagues [24]. In a cross-sectional, multicentric, observational study conducted in Turkey on patients with psoriasis on chronic immunosuppressive treatment, worsening psoriasis was reported in 24.2% (442/1827) of patients. Patients receiving conventional drugs had a significantly higher frequency of worsened psoriasis than patients receiving anti-TNF-α or anti-IL drugs [25]. Pirro and colleagues, in their telephone survey, analyzed the impact of COVID-19 pandemic in a cohort of psoriatic patients on biologic treatment. Sixty-three of 226 (27.9%) participants exhibited worsening psoriasis (mainly because of treatment interruption). Worsening psoriasis significantly correlated with Hospital Anxiety and Depression Scale (HADS) anxiety, HADS depression, Brief Resilience Scale (BRS), and Perceived Stress Scale (PSS) abnormal scores [26]. Polat et al. reported that 15 out of 23 patients (17 of whom treated with systemic therapy, including 9 patients on biologics) diagnosed with COVID-19 experienced a psoriasis exacerbation after the viral disease and only one (in conjunction) with the SARS-CoV-2 infection [27]. As reported in a recent survey of psoriasis patients on biologics in the northeastern of France during COVID-19 pandemic, a psoriasis flare-up was observed by 15 (3%, 5 of whom reported discontinuation of biologics) out of 485 contacted patients [28]. A similar survey conducted in a single center in Italy reported a worsening psoriasis (from Psoriasis Area Severity Index (PASI) 90 to PASI 75) in 9 out of 27 (33.3%) patients who discontinued their biologic treatment [29]. Recently, Mahil and colleagues investigated the factors associated with worsening psoriasis during the COVID-19 outbreak. The researchers included 4043 psoriatic patients (from 86 countries) in their global cross-sectional study. Nearly 43% (1728) of these patients reported worsening psoriasis in the pandemic. Associations with worsening psoriasis were observed for poor mental health (anxiety or depression), female gender, obesity, and shielding. Interestingly, targeted (and also systemic) therapy use (TNF-α inhibitors, IL-17 inhibitors, IL-23 inhibitors, and apremilast) was inversely associated with worsening psoriasis, with an estimated odds ratio (OR) of 0.49 [30].

**Figure 1:**
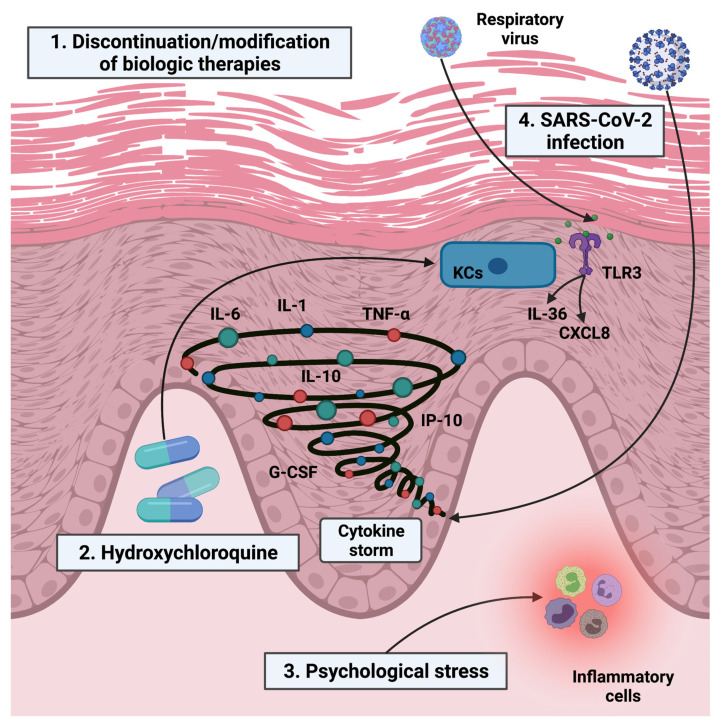
Possible pathogenetic mechanisms at the basis of psoriasis exacerbation. Created with BioRender.com. (November 9th, 2021) 1. Discontinuation or modification (lengthening of the dosing interval) of biologic therapies. 2. Hydroxychloroquine (HCQ). As demonstrated by Wolf et al. in their in vitro study, HCQ induced hyperproliferation and irregular keratinization on the cultured skin of psoriatic patients. These changes in the epidermal morphology aimed at barrier restoration following an initial break in the epidermal barrier (probably due to HCQ inhibiting effect on epidermal transglutaminase activity). Thus, this epidermal proliferation is probably sufficient to trigger induction or worsening of psoriasis [45]. In addition, HCQ promotes interleukin (IL)-17 production via p38-dependent IL-23 release, which results in keratinocyte growth and differentiation [46]. 3. Psychological stress. As reported by Stewart and colleagues, psychological stress may increase trafficking of inflammatory cells to the skin and potentiate neurogenic inflammation [49]. 4. SARS-CoV-2 infection. Sbidian et al. hypothesized that overproduction of several cytokines and chemokines, such as IL-36 and CXCL8, after stimulation with polyinosinic-polycytidylic acid (a TLR3 agonist that mimics RNA of respiratory viruses), might be linked to pathophysiology of psoriasis flare-ups [52]. Abbreviations: IL, interleukin; TNF-α, tumour necrosis factor alpha; G-CSF, granulocyte-colony stimulating factor; IP-10, interferon-γ inducible protein 10; SARS-CoV-2, severe acute respiratory syndrome coronavirus 2; TLR3, Toll-like receptor 3; CXCL8, chemokine (C-X-C motif) ligand 8; and KCs, keratinocytes.

The exacerbation of psoriasis may be explained by several conditions (Figure 1) [31]. First, psoriasis flare-ups might be attributed to the discontinuation of treatments [12,27,28,29]. Furthermore, non-adherence to biological therapy could determine not only the exacerbation of psoriasis but also the development of antibodies following suspension and then reintroduction of biologic drug, which consequently might result in switching toward more expensive drugs due to loss of efficacy after the interrumption [32,33]. A postal survey conducted in Germany during the time of COVID-19 confirmed that missed appointments and therapy changes (paused, stopped, switched, altered dosing regime, and not specified) because of the pandemic were associated with more frequent psoriasis aggravation [34]. A multicenter retrospective study conducted in Canada found that the rate of patient-driven biologic discontinuation during the peak of COVID-19 cases was 1.1% (23/2095), and discontinuation of treatment led to a flare of psoriasis in five patients [35]. Carugno and colleagues, in a high-epidemic area in Italy, observed a worsening of psoriasis, after stopping therapy on their own, in 3 (2.3%) out of 130 patients on biologics (without suspected COVID-19 symptoms) [36]. As reported by Mahil et al., 284 (18.4%) out of 1541 psoriatic patients receiving standard systemic or targeted therapies (including biologics) were non-adherent to treatment. The researchers found that non-adherence was associated with worsening psoriasis (OR, 2.90) [30]. An observational, multicentric study including 12,807 patients with moderate-to-severe psoriasis undergoing treatment with any biological agent showed that, autonomously, 328 patients (2.6%) stopped treatment and 185 (1.4%) patients lengthened time intervals for treatment administration [37]. Rob and colleagues, in their multicenter study in the Czech Republic during the COVID-19 pandemic national lockdown, reported that patient concerns about the safety of their treatment were significantly more common in patients treated with biologics (40.7%) than in those treated with conventional systemic therapy (21.3%) and topical therapy (10.9%) [38]. Bragazzi et al., in an Italian multicenter survey during the “red zone”, found (at the multivariate logistic regression analyses) that in patients treated with biologics (plaque psoriasis, atopic dermatitis, or hidradenitis suppurativa) independent predictors of continuation vs. discontinuation and modification vs. no modification (of biologic therapies) were only the knowledge of COVID-19-related epidemiology and COVID-19-related preventive measures, respectively [39]. Wang and colleagues, in their web-based survey including 926 valid questionnaires, reported that 68.5% of participants were non-adherent to treatment (31.2% for less than two weeks and 37.3% for more than two weeks) and non-adherence rate was lower among patients receiving biological treatment (37.3%) compared to systemic (63.7%) and topical treatment (71.2%). Furthermore, non-adherence to treatment was significantly associated not only with psoriasis exacerbation but also with perceived stress, symptoms of depression, and anxiety. Interestingly, perceived stress was positively associated with non-adherence to biological therapy, whereas depression symptoms were associated with non-adherence to systemic and topical treatments [40]. In line with these findings, Polat Ekinci and colleagues found that, out of a total of 133 psoriatic patients on biological therapy included in their survey study, 52 (39%) of participants interrupted their therapy. The incidence of psoriais activation (new psoriatic skin lesions and/or activation of psoriatic arthritis) was 6.2%, 33.3%, and 42.1% in adhered patients and short- and long-term treatment suspension groups, respectively [41]. In a recent survey assessing psoriasis patients’ understanding of biologic use during the COVID-19 outbreak, 56.9% and 56.9% of participants answered they would stop their biologic or reduce dosage, respectively [42]. Second, psoriasis exacerbation might be attributed to COVID-19 treatments [31]. Although available evidence does not support the use of hydroxychloroquine (HCQ) in the prevention or treatment of COVID-19 (also in patients with psoriatic disease outside of a clinical trial) [7,43], it was used in the early COVID-19 pandemic [9,10,11,15,16]. Sachdeva and colleagues, in their recent systematic review, evaluated HCQ effects on psoriasis. The research found that, out of a total of 18 patients included in the study, nine (50.0%) experienced a psoriasis onset, five (27.8%) an exacerbation of psoriatic symptoms, and four (22.2%) a relapse of psoriasis after HCQ treatment [44]. Several potential mechanisms may be implicated in psoriatic flare induced by HCQ (Figure 1) [44,45,46]. In addition, oral steroids (used for hospitalized patients infected with SARS-CoV-2 meeting specific criteria) may be responsible for the induction or worsening of erythrodermic and pustular psoriasis [47,48]. Third, psychological stress (as a direct consequence of the COVID-19 pandemic) may be a major risk factor for psoriasis flare-up [49]. Kuang et al. reported that outdoor activity restriction and income loss were positively associated with the exacerbation of psoriasis, stress, and symptoms of anxiety and depression [50]. Lastly, SARS-CoV-2 infection may play a direct role in worsening psoriasis. Zika, chikungunya, and dengue infections have already been reported as exacerbating factors of psoriasis in patients on biological therapy [51]. Rather recently, Sbidian and colleagues documented that 21 out of 25 patients (14 of whom treated with biological agents) presenting with a psoriasis flare following respiratory tract infection had at least one positive molecular viral testing on nasopharyngeal swab (Rhinovirus and subtypes of Coronavirus were the most frequently detected viral pathogens). Only one patient reported delayed ustekinumab injection and psychological stress as possible triggers of psoriasis flare-up. The researchers also suggested a possible mechanism of action underlying the exacerbation of psoriasis (Figure 1) [52]. In addition, patients with COVID-19 have a state of hyperinflammation: activation of immune cell subsets results in the uncontrolled expression of massive inflammatory cytokine cascade (cytokine storm), which can trigger psoriasis exacerbation [31,53].

#### 3.1.2. Cutaneous Manifestations and Anti-SARS-CoV2 Immunization

Three anti-SARS-CoV2 vaccines have been authorized for use both in the European Union by the European Medicines Agency (EMA; Amsterdam, The Netherlands) and in the United State by the US Food and Drug Administration (FDA): BNT162b2 (Pfizer-BioNTech, New York, NY, USA/Mainz, Germany), mRNA-1273 (Moderna; Cambridge, MA, USA), and Ad26.COV2.S (Janssen; Janssen-Cilag International NV; Beerse, Belgium). The first two are mRNA-based vaccines, whereas the Ad26.COV2.S is a viral (attenuated human adenovirus serotype 26, Ad26) vector-based vaccine. A further adenovirus-based vaccine, ChAdOx1 (Oxford-AstraZeneca; Cambridge, UK), has only been approved by the EMA [54,55]. Although there is a need for education about vaccines for psoriatic patients while on biologics during the COVID-19 outbreak [56], Sotiriou and colleagues, in their study examining the intention of COVID-19 vaccination among psoriatic patients, found that treatment with biologics appeared to contribute positively to COVID-19 vaccination [57]. Cutaneous reactions after mRNA-based COVID-19 vaccine (Moderna and Pfizer) administration were described in a registry-based study of 414 cases by McMahon et al. The researchers reported two cases of psoriasis flare-up among these 414 patient records (neither the skin lesion description nor the type of mRNA-based vaccine are reported) [58]. Other cases of psoriasis exacerbation after different types of anti-SARS-CoV-2 vaccines have been recently described (Table 2). All patients had a history of psoriasis, but none were on biologics [59,60,61,62,63,64,65,66,67]. In addition, four cases of new-onset psoriasis (all patients had no personal history of psoriasis) following anti-SARS-CoV-2 vaccination have also been described in the literature: (i) a 66-year-old female who developed generalized pustular psoriasis 3 weeks after the first dose of the Oxford-AstraZeneca COVID-19 vaccine, (ii) a 79-year-old female who presented de novo guttate psoriasis 10 days after receiving the first injection with the Pfizer-BioNTech (New York, NY, USA/Mainz, Germany) vaccine (the second dose led again to a psoriasis flare-up), (iii) a 65-year-old male with scaly erythematous papules and plaques over trunk and extremities that started 10 days after he received the second dose of Oxford-AstraZeneca/Covishield vaccine and (iv) a 72-year-old male who developed new-onset generalized erythematous desquamating plaques 6 days after receiving the second dose of the mRNA-1273 (Moderna) vaccine [64,66,68,69]. Watad and colleagues reported a case of a 36-year-old female with psoriasis since childhood who developed a new-onset immune-mediated disease (dactylitis, joint pain, stiffness, and tightness of fingers associated with erythematous macules over palmar surface and chilblains like lesions on fingers) 10 days after the first dose of mRNA-1273 (Moderna) vaccine [70]. Data on (possible) skin manifestations after COVID-19 vaccination in psoriatic patients treated with biologics available in the literature are scarce. Recently, Skroza and colleagues investigated the impact (if any) of COVID-19 vaccination on the course and the severity of psoriatic disease. For this purpose, their study included 436 patients with moderate-to-severe psoriasis in treatment with biologics, 78 of whom had received the Pfizer-BioNTech mRNA vaccine (vaccinee group). None of patients experienced adverse drug reactions (ADRs) after vaccination. The average PASI at baseline and after 24-week treatment with biologics decreased by 74.13% and 73.4% in the general (vaccinated and non-vaccinated subjects) and in vaccinee groups, respectively. The authors hypothesized that this slight but not significant difference in the vaccinee group might be explained by the 10-day delay in the scheduled administration of biologics after vaccination [71]. In line with these findings, Damiani et al. did not report any psoriasis flare-up or vaccine-related cutaneous manifestation in four psoriatic patients (one female and three males) under biologics (two secukinumab, one ixekizuamb, andone risankizumab) that underwent mRNABNT162b2 (Pfizer-BioNTech) vaccine, suggesting that mRNA-based COVID-19 vaccines do not trigger psoriasis flares in psoriatic patients undergoing target therapies (biologics) [72]. Conversely, Megna and colleagues observed 11 cases (eight males) of psoriasis exacerbation after COVID-19 vaccination (seven with Pfizer-BioNTech, three with AstraZeneca-Oxford, and one with Moderna vaccine). Interestingly, the researchers reported six cases (54.5%) of these psoriasis flares in subjects under biologic treatment [73]. Recently, Musumeci and colleagues described only an exacerbation of psoriasis (out of 50 patients on biologics after the first and second doses of Pfizer-BioNTech or Moderna vaccines) after Pfizer-BioNTech vaccine in a patient treated with infliximab biosimilar [74]. The possible increase of IL-2, IL-12, TNF-α, and interferon (IFN)-γ levels after mRNA COVID-19 vaccination might be the rationale for new onset psoriasis and flares after mRNA COVID-19 vaccine administration [66]. Recently, Talamonti and Galluzzo observed no serious adverse events related to vaccination against COVID-19 in 369 patients with psoriasis receiving therapy with anti-IL agents [75].

#### 3.1.3. (Other) Cutaneous Manifestation

Concerning other possible cutaneous manifestations in psoriatic patients undergoing biological therapy during COVID-19 pandemic, to the best of our knowledge, at the time of writing, only one case has been reported. Carugno and colleagues described their experience with a 69-year-old male psoriatic patient (psoriasis and psoriatic arthritis) treated with secukinumab who developed an erythemato-oedematous morbilliform rash associated with a flare-up of his psoriasis following a mild form of COVID-19. The morbilliform rash occurred about 8 weeks after the last secukinumab injection [77].

### 3.2. Hidradenitis Suppurativa

HS is a chronic, (auto)inflammatory disease primarily affecting apocrine gland-rich areas of the body [78]. Even though it is known that there is an overlap between severe COVID-19 risk factors and HS comorbidities (history of smoking, hypertension, diabetes, cardiovascular complications, obesity, and nonalcoholic fatty liver disease) [79,80], real-life data suggested otherwise, with neither an increased susceptibility to SARS-CoV-2 infection nor a more severe course of COVID-19 (than that of the general population) reported among HS patients (even on biologic treatment) [81,82,83,84,85,86]. The younger age of HS patients might be a protective factor against severe COVID-19 [86]. In view of this, experts of the European Hidradenitis Suppurativa Foundation e.V. (EHSF) agreed that treatment with the TNF-α inhibitor, adalimumab (the only biologic agent approved by both FDA and EMA for moderate-to-severe HS), is probably not associated with an increased risk for/a more severe course of COVID-19 [87]. Thus, the second key question of this short review was as follows: could the COVID-19 pandemic have an impact on skin manifestations of HS patients undergoing biological therapy? Freeman et al., with the aim of characterizing the spectrum of COVID-19-associated cutaneous manifestations, reported (among 171 subjects with new-onset dermatologic symptoms and laboratory-confirmed COVID-19) one case of pernio-like lesions and one case of vesicular eruption in two patients with previous HS (HS treatment was not specified) [88]. However, although only few data on this topic exist, it is conceivable that COVID-19 pandemic might lead to a worsening of skin manifestations in HS patients undergoing biological therapy. Indeed, discontinuation or modification (lengthening of the dosing interval) of biologic therapies and body weight gain associated with hypovitaminosis D (due to reduced physical activities, increased intake of calories, and lack of sun exposure, because of self-isolation/quarantine or restriction measures) might favor HS flare-ups more frequently than in the COVID-19 pre-pandemic period [89,90,91]. Furthermore, the reduction or suppression of clinical response in HS patients, once adalimumab is reintroduced (after discontinuation), might be related to the development of anti-drug-antibodies [92]. Price and colleagues, in their study examining the influence of COVID-19 pandemic on HS care, reported that 61.2% (205/355) of the respondents (to an anonymous questionnaire distributed via HS support group Facebook pages) experienced increased HS flares. According to the authors of the article, this might be related to reported weight gain, increased smoking, and financial stress during the COVID-19 pandemic. In addition, more than half of respondents (51.2%, 171/334) avoided going to the emergency room/urgent care for HS flares. Interestingly, only 11.3% (9/80) of the respondents (despite concerns about COVID-19) stopped using their HS injectable medications [93]. On the other hand, Caposiena Caro et al. described the case of a 37-year-old female with a very severe HS, non-responsive to several medical approaches (including adalimumab, anakinra, etanercept, infliximab, and ustekinumab), who progressively improved her HS signs and symptoms after COVID-19 infection. The researchers suggested two intriguing hypotheses for this “paradoxical” improvement: (i) an immune response resetting as a consequence of the massive immune response against SARS-CoV-2 and (ii) a possible shift of the immune response initiated by SARS-CoV-2 (which has acted as a second antigen) [94]. Finally, based on our experience, it is conceivable that wearing loose-fitting clothing (avoiding belts, for example) during restriction measures or smart working might have avoided mechanical friction (which promotes follicular occlusion in HS patients), consequently leading to an improvement of HS skin manifestations [78]. Concerning anti-SARS-CoV-2 immunization and HS, although vaccination is recommended, further studies are needed to determine potential vaccination effects (if any) on HS (and vice versa) [87].

## 4. Conclusions

The COVID-19 outbreak has raised significant concerns about the management of moderate to-severe forms of psoriasis and HS. In the light of available data, adherence to biological therapy is recommended in this subset of patients. Nevertheless, dermatologists should be aware that COVID-19 pandemic (in all its facets) might have an impact on skin manifestations of psoriatic and HS patients undergoing biological therapy, making the management of these fragile subjects more challenging.

## Figures and Tables

**Table 1:** A summary of cases reporting psoriasis exacerbation following anti-SARS-CoV-2 infection.

Age Gender	COVID-19 Diagnosis	Psoriasis Flare-Up Manifestation	COVID-19 Treatments	Psoriasis Flare-Up Treatments	Ref.
73-year-old male	PCR Chest CT	Diffuse erythematous scaly plaques progressing to erythroderma	HCQ (200 mg b.i.d.) + lopinavir/ritonavir combination (b.i.d.) + acetaminophen (500 mg q.i.d.)	CsA (100 mg QD)	[9]
71-year-old female	Not reported	Exacerbation of silver-scaled psoriatic plaques	HCQ (400 mg b.i.d. on the first day, then 200 mg b.i.d.) + oseltamivir (75 mg b.i.d.)	Not reported	[10]
48-year-old female	PCR Chest CT	Active psoriatic lesions on scalp, trunk, and extremities. PASI: 24	HCQ + azithromycin + oseltamivir + inhaled ipratropium and budesonide	No active drug	[11]
45-year-old male Drug addict	PCR +blood culture positive for SA	Erythroderma	Cloxacillin + vancomycin + meropenem + enoxaparin + methadone	CsA (100 mg b.i.d.) + PSL (10 mg QD) + ACT (35 mg QD then reduced to 10 mg QD)	[12]
38-year-old male	PCR	Guttate psoriasis	Not reported	BMZ 0.025% cream (b.i.d.)	[13]
44-year-old male	Not reported	Widespread plaque psoriasis	Not reported	Not reported	[14]
60-year-old male	PCR Chest CT	Generalized pustular psoriasis	HCQ + naproxen + meropenem + linezolid + vitamin D3 + heparin + intravenous pulse MPSL + PSL at discharge (30 mg QD)	ACT (25 mg QD)	[15]
47-year-old female	PCR Chest CT	Pustular psoriasis	HCQ (400 mg b.i.d. on the first day and then 200 mg b.i.d. for the next 4 days)	Not reported	[16]
21-year-old female	Not reported	Guttate psoriasis	Not reported	Halobetasol 0.5% lotion	[17]
32-year-old female	PCR Chest CT	Generalized pustular psoriasis	HCQ + azithromycin	PSL (50 mg QD) + CsA (100 mg b.i.d.) + clobetasol 0.05%	[18]
72-year-old female	PCR	Generalized pustular psoriasis	Azithromycin + paracetamol	ACT (50 mg QD) + infliximab IV (5 mg/kg body weight)	[19]

Abbreviations: PCR, polymerase chain reaction; CT, computed tomography; HCQ, hydroxychloroquine; CsA, cyclosporine; SA, Staphylococcus aureus; PSL, prednisolone; ACT, acitretin; BMZ, betamethasone; and MPSL, methylprednisolone.

**Table 2:** A summary of cases reporting psoriasis exacerbation following anti-SARS-CoV-2 vaccination.

Age Gender	COVID-19 Vaccine	Dose	Days	Psoriasis Flare-Up Type	Reference
(55–82) 9 females (49–83) 5 males	5 ChAdOx1 (Oxford-AstraZeneca) 3 BNT162b2 (Pfizer-BioNTech) 1 mRNA-1273 (Moderna) 3 BNT162b2 (Pfizer-BioNTech) 2 ChAdOx1 (Oxford-AstraZeneca)	2 2 2 2 1	3–13 5–7 10 7–10 22–32	4 Plaque psoriasis 1 Guttate psoriasis Plaque psoriasis Plaque psoriasis Plaque psoriasis Plaque psoriasis	[59]
46-year-old male	BNT162b2 (Pfizer-BioNTech)	2	5	Plaque psoriasis	[60]
40-year-old male	BNT162b2 (Pfizer-BioNTech)	1	5	Acute generalized pustular psoriasis	[61]
72-year-old male	CoronaVac * (Sinovac Biotech)	1	4	Generalized pustular psoriasis	[62]
51-year-old male 52-year-old male	BNT162b2 (Pfizer-BioNTech) CoronaVac * (Sinovac Biotech)	1 2	NR 30	Plaque psoriasis Plaque psoriasis	[63]
56-year-old female	Oxford-AstraZeneca (Covishield)	2	2	Plaque psoriasis	[64]
34-year-old female	Oxford-AstraZeneca	1	14	Plaque psoriasis	[65]
30-year-old female	mRNA-1273 (Moderna)	1	10	Plaque psoriasis	[66]
65-year-old male	BNT162b2 (Pfizer-BioNTech)	1	7	Plaque psoriasis	[67]

* CoronaVac has received World Health Organization (WHO) emergency use authorization and is currently approved in 32 countries [76].

## References

[1] Hu B., Guo H., Zhou P., Shi Z.-L. (2021). Characteristics of SARS-CoV-2 and COVID-19. Nat. Rev. Microbiol..

[2] Genovese G., Moltrasio C., Berti E., Marzano A.V. (2020). Skin manifestations associated with COVID-19: Current knowledge and future perspectives. Dermatology.

[3] Seirafianpour F., Sodagar S., Pour Mohammad A., Panahi P., Mozafarpoor S., Almasi S., Goodarzi A. (2020). Cutaneous manifestations and considerations in COVID-19 pandemic: A systematic review. Dermatol. Ther..

[4] Armstrong A.W., Read C. (2020). Pathophysiology, clinical presentation, and treatment of psoriasis: A review. JAMA.

[5] Dattola A., Silvestri M., Tamburi F., Amoruso G.F., Bennardo L., Nisticò S.P. (2020). Emerging role of anti-IL23 in the treatment of psoriasis: When humanized is very promising. Dermatol. Ther..

[6] Amoruso G., Nisticò S., Iannone L., Russo E., Rago G., Patruno C., Bennardo L. (2021). Ixekizumab may improve renal function in psoriasis. Healthcare.

[7] Gelfand J.M., Armstrong A.W., Bell S., Anesi G.L., Blauvelt A., Calabrese C., Dommasch E.D., Feldman S.R., Gladman D., Kircik L. (2021). National Psoriasis Foundation COVID-19 Task Force guidance for management of psoriatic disease during the pandemic: Version 2—Advances in psoriatic disease management, COVID-19 vaccines, and COVID-19 treatments. J. Am. Acad. Dermatol..

[8] El-Komy M.H., Abdelnaby A., El-Kalioby M. (2021). How does COVID-19 impact psoriasis practice, prescription patterns, and healthcare delivery for psoriasis patients? A cross-sectional survey study. J. Cosmet. Dermatol..

[9] Nasiri S., Araghi F., Tabary M., Gheisari M., Mahboubi-Fooladi Z., Dadkhahfar S. (2020). A challenging case of psoriasis flare-up after COVID-19 infection. J. Dermatol. Treat..

[10] Kutlu Ö., Metin A. (2020). A case of exacerbation of psoriasis after oseltamivir and hydroxychloroquine in a patient with COVID-19: Will cases of psoriasis increase after COVID-19 pandemic?. Dermatol. Ther..

[11] Ozaras R., Berk A., Ucar D.H., Duman H., Kaya F., Mutlu H. (2020). Covid-19 and exacerbation of psoriasis. Dermatol. Ther..

[12] Ghalamkarpour F., Pourani M.R., Abdollahimajd F., Zargari O. (2020). A case of severe psoriatic erythroderma with COVID-19. J. Dermatol. Treat..

[13] Gananandan K., Sacks B., Ewing I. (2020). Guttate psoriasis secondary to COVID-19. BMJ Case Rep..

[14] Al Abadie M.S. (2020). COVID-19 infection cause moderate-severe psoriasis flare up. Eur. J. Med. Health Sci..

[15] Dadras M.S., Diab R., Ahadi M., Abdollahimajd F. (2020). Generalized pustular psoriasis following COVID-19. Dermatol. Ther..

[16] Shakoei S., Ghanadan A., Hamzelou S. (2020). Pustular psoriasis exacerbated by COVID-19 in a patient with the history of psoriasis. Dermatol. Ther..

[17] Agarwal A., Tripathy T., Kar B.R. (2021). Guttate flare in a patient with chronic plaque psoriasis following COVID-19 infection: A case report. J. Cosmet. Dermatol..

[18] Miladi R., Janbakhsh A., Babazadeh A., Aryanian Z., Ebrahimpour S., Barary M., Sio T.T., Wollina U., Goldust M., Afshar Z.M. (2021). Pustular psoriasis flare-up in a patient with COVID-19. J. Cosmet. Dermatol..

[19] Samotij D., Gawron E., Szczęch J., Ostańska E., Reich A. (2021). Acrodermatitis continua of Hallopeau evolving into generalized pustular psoriasis following COVID-19: A case report of a successful treatment with infliximab in combination with acitretin. Biol. Targets Ther..

[20] Mathieu R.J., Cobb C.B., Telang G.H., Firoz E.F. (2020). New-onset pustular psoriasis in the setting of severe acute respiratory syndrome coronavirus 2 infection causing coronavirus disease 2019. JAAD Case Rep..

[21] Rouai M., Rabhi F., Mansouri N., Jaber K., Dhaoui R. (2021). New-onset guttate psoriasis secondary to COVID-19. Clin. Case Rep..

[22] Mroz M., Mućka S., Miodońska M., Ziolkowska D., Hadas E., Bożek A. (2021). Influence of SARS-CoV-2 virus infection on the course of psoriasis during treatment with biological drugs. Medicina.

[23] Yalici-Armagan B., Tabak G.H., Dogan-Gunaydin S., Gulseren D., Akdogan N., Atakan N. (2021). Treatment of psoriasis with biologics in the early COVID-19 pandemic: A study examining patient attitudes toward the treatment and disease course. J. Cosmet. Dermatol..

[24] Filippi F., Loi C., Evangelista V., Bardazzi F. (2020). COVID-19 era: A chance to learn something new about monitoring psoriatic patients in biological therapy. Dermatol. Ther..

[25] Kartal S.P., Çelik G., Yılmaz O., Solak E., Gül B.D., Üstünbaş T.K., Gönülal M., Baysak S., Yüksel E.I., Ünlü B. (2021). The impact of COVID-19 pandemic on psoriasis patients, and their immunosuppressive treatment: A cross-sectional multicenter study from Turkey. J. Dermatol. Treat..

[26] Pirro F., Caldarola G., Chiricozzi A., Tambone S., Mariani M., Calabrese L., D’Urso D.F., De Simone C., Peris K. (2020). The impact of COVID-19 pandemic in a cohort of Italian psoriatic patients treated with biological therapies. J. Dermatol. Treat..

[27] Polat A.K., Topal I.O., Karadag A.S., Aksoy H., Aksu A.E.K., Ozkur E., Akbulut T.O., Demir F.T., Engin B., Uzuncakmak T.K. (2020). The impact of COVID-19 in patients with psoriasis: A multicenter study in Istanbul. Dermatol. Ther..

[28] Gallais-Serezal I., Puzenat E., Moreau J., Dresco F., Pelletier F., Nardin C., Aubin F. (2021). A survey of psoriasis patients on biologics during COVID-19: A high-epidemic area experience—Franche Comté, France. Eur. J. Dermatol..

[29] Burlando M., Carmisciano L., Cozzani E., Parodi A. (2020). A survey of psoriasis patients on biologics during COVID-19: A single centre experience. J. Dermatol. Treat..

[30] Mahil S., Yates M., Yiu Z., Langan S., Tsakok T., Dand N., Mason K., McAteer H., Meynell F., Coker B. (2021). Describing the burden of the COVID-19 pandemic in people with psoriasis: Findings from a global cross-sectional study. J. Eur. Acad. Dermatol. Venereol..

[31] Aram K., Patil A., Goldust M., Rajabi F. (2021). COVID-19 and exacerbation of dermatological diseases: A review of the available literature. Dermatol. Ther..

[32] Nogueira M., Vender R., Torres T. (2020). Psoriasis, biologic therapy, and the pandemic of the 21st century. Drugs Context.

[33] Megna M., Napolitano M., Patruno C., Fabbrocini G. (2020). Biologics for psoriasis in COVID-19 era: What do we know?. Dermatol. Ther..

[34] Ninosu N., Roehrich F., Diehl K., Peitsch W.K., Schaarschmidt M.-L. (2021). Psoriasis care during the time of COVID-19: Real-world data on changes in treatments and appointments from a German university hospital. Eur. J. Dermatol..

[35] Georgakopoulos J.R., Mufti A., Vender R., Yeung J. (2020). Treatment discontinuation and rate of disease transmission in psoriasis patients receiving biologic therapy during the COVID-19 pandemic: A Canadian multicenter retrospective study. J. Am. Acad. Dermatol..

[36] Carugno A., Gambini D.M., Raponi F., Vezzoli P., Locatelli A.G.C., Di Mercurio M., Test E.R., Sena P. (2020). COVID-19 and biologics for psoriasis: A high-epidemic area experience—Bergamo, Lombardy, Italy. J. Am. Acad. Dermatol..

[37] Talamonti M., Galluzzo M., Chiricozzi A., Quaglino P., Fabbrocini G., Gisondi P., Marzano A., Potenza C., Conti A., Parodi A. (2020). Management of biological therapies for chronic plaque psoriasis during COVID-19 emergency in Italy. J. Eur. Acad. Dermatol. Venereol..

[38] Rob F., Hugo J., Tivadar S., Boháč P., Gkalpakiotis S., Vargová N., Arenbergerová M., Hercogová J. (2020). Compliance, safety concerns and anxiety in patients treated with biologics for psoriasis during the COVID-19 pandemic national lockdown: A multicenter study in the Czech Republic. J. Eur. Acad. Dermatol. Venereol..

[39] Bragazzi N., Riccò M., Pacifico A., Malagoli P., Kridin K., Pigatto P., Damiani G. (2020). COVID-19 knowledge prevents biologics discontinuation: Data from an Italian multicenter survey during RED-ZONE declaration. Dermatol. Ther..

[40] Wang Q., Luo Y., Lv C., Zheng X., Zhu W., Chen X., Shen M., Kuang Y. (2020). Nonadherence to treatment and patient-reported outcomes of psoriasis during the COVID-19 epidemic: A web-based survey. Patient Prefer. Adherence.

[41] Ekinci A.P., Pehlivan G., Gökalp M.O. (2020). Surveillance of psoriatic patients on biologic treatment during the COVID-19 pandemic: A single-center experience. Dermatol. Ther..

[42] Pandher K., Porter C., Patel H., Huang W., Feldman S. (2020). Understanding views of patients on biologics for psoriasis amid the COVID-19 pandemic. J. Eur. Acad. Dermatol. Venereol..

[43] Jorge A. (2020). Hydroxychloroquine in the prevention of COVID-19 mortality. Lancet Rheumatol..

[44] Sachdeva M., Mufti A., Maliyar K., Lytvyn Y., Yeung J. (2020). Hydroxychloroquine effects on psoriasis: A systematic review and a cautionary note for COVID-19 treatment. J. Am. Acad. Dermatol..

[45] Wolf R., Lo Schiavo A., Lombardi M.L., De Angelis F., Ruocco V. (1999). The in vitro effect of hydroxychloroquine on skin morphology in psoriasis. Int. J. Dermatol..

[46] Said A., Bock S., Lajqi T., Müller G., Weindl G. (2014). Chloroquine promotes IL-17 production by CD4^+^ T cells via p38-dependent IL-23 release by monocyte-derived Langerhans-like cells. J. Immunol..

[47] Elston G.E., Carr R.A., Charles-Holmes R. (2006). Precipitation of generalized pustular psoriasis by prednisolone. Clin. Exp. Dermatol..

[48] Rendo M., Boster J., Dalton S.R., Yun H. (2019). An uncommon presentation of erythrodermic psoriasis in a patient without a history of psoriasis. Cureus.

[49] Stewart T.J., Tong W., Whitfeld M.J. (2018). The associations between psychological stress and psoriasis: A systematic review. Int. J. Dermatol..

[50] Kuang Y., Shen M., Wang Q., Xiao Y., Lv C., Luo Y., Zhu W., Chen X. (2020). Association of outdoor activity restriction and income loss with patient-reported outcomes of psoriasis during the COVID-19 pandemic: A web-based survey. J. Am. Acad. Dermatol..

[51] Araujo K.M., Bressan A.L., Azulay-Abulafia L. (2020). Zika, chikungunya, and dengue infections as exacerbating factors of psoriasis in patients receiving biological therapy. Int. J. Dermatol..

[52] Sbidian E., Madrange M., Viguier M., Salmona M., Duchatelet S., Hovnanian A., Smahi A., Le Goff J., Bachelez H. (2019). Respiratory virus infection triggers acute psoriasis flares across different clinical subtypes and genetic backgrounds. Br. J. Dermatol..

[53] Freeman T., Swartz T.H. (2020). Targeting the NLRP3 inflammasome in severe COVID-19. Front. Immunol..

[54] Lythgoe M.P., Middleton P. (2021). Comparison of COVID-19 vaccine approvals at the US Food and drug administration, European medicines agency, and health Canada. JAMA Netw. Open.

[55] Diotallevi F., Campanati A., Radi G., Martina E., Rizzetto G., Barbadoro P., D’Errico M.M., Offidani A. (2021). Vaccines against SARS-CoV-2 in psoriasis patients on immunosuppressive therapy: Implications of vaccination nationwide campaign on clinical practice in Italy. Dermatol. Ther..

[56] Le H., Vender R.B. (2021). A psoriatic patient-based survey on the understanding of the use of vaccines while on biologics during the COVID-19 pandemic. J. Cutan. Med. Surg..

[57] Sotiriou E., Bakirtzi K., Papadimitriou I., Paschou E., Vakirlis E., Lallas A., Ioannides D. (2021). COVID-19 vaccination intention among patients with psoriasis compared with immunosuppressed patients with other skin diseases and factors influencing their decision. Br. J. Dermatol..

[58] McMahon D.E., Amerson E., Rosenbach M., Lipoff J.B., Moustafa D., Tyagi A., Desai S.R., French L.E., Lim H.W., Thiers B.H. (2021). Cutaneous reactions reported after Moderna and Pfizer COVID-19 vaccination: A registry-based study of 414 cases. J. Am. Acad. Dermatol..

[59] Sotiriou E., Tsentemeidou A., Bakirtzi K., Lallas A., Ioannides D., Vakirlis E. (2021). Psoriasis exacerbation after COVID-19 vaccination: A report of 14 cases from a single centre. J. Eur. Acad. Dermatol. Venereol..

[60] Krajewski P., Matusiak Ł., Szepietowski J. (2021). Psoriasis flare-up associated with second dose of Pfizer-BioNTech BNT16B2b2 COVID-19 mRNA vaccine. J. Eur. Acad. Dermatol. Venereol..

[61] Perna D., Jones J., Schadt C.R. (2021). Acute generalized pustular psoriasis exacerbated by the COVID-19 vaccine. JAAD Case Rep..

[62] Onsun N., Kaya G., Işık B.G., Güneş B. (2021). A generalized pustular psoriasis flare after CoronaVac COVID-19 vaccination: Case report. Health Promot. Perspect..

[63] Bostan E., Elmas L., Yel B., Yalici-Armagan B. (2021). Exacerbation of plaque psoriasis after inactivated and BNT162b2 mRNA COVID-19 vaccines: A report of two cases. Dermatol. Ther..

[64] Nagrani P., Jindal R., Goyal D. (2021). Onset/flare of psoriasis following the ChAdOx1 nCoV-19 Corona virus vaccine (Oxford-AstraZeneca/Covishield): Report of two cases. Dermatol. Ther..

[65] Fang W., Chiu L., Hu S.C. (2021). Psoriasis exacerbation after first dose of AstraZeneca coronavirus disease 2019 vaccine. J. Dermatol..

[66] Pesqué D., Lopez-Trujillo E., Marcantonio O., Giménez-Arnau A.M., Pujol R.M. (2021). New-onset and exacerbations of psoriasis after mRNA COVID-19 vaccines: Two sides of the same coin?. J. Eur. Acad. Dermatol. Venereol..

[67] Mieczkowska K., Kaubisch A., McLellan B.N. (2021). Exacerbation of psoriasis following COVID-19 vaccination in a patient previously treated with PD-1 inhibitor. Dermatol. Ther..

[68] Elamin S., Hinds F., Tolland J. (2021). De novo generalized pustular psoriasis following Oxford-AstraZeneca COVID-19 vaccine. Clin. Exp. Dermatol..

[69] Lehmann M., Schorno P., Hunger R., Heidemeyer K., Feldmeyer L., Yawalkar N. (2021). New onset of mainly guttate psoriasis after COVID-19 vaccination: A case report. J. Eur. Acad. Dermatol. Venereol..

[70] Watad A., De Marco G., Mahajna H., Druyan A., Eltity M., Hijazi N., Haddad A., Elias M., Zisman D., Naffaa M. (2021). Immune-mediated disease flares or new-onset disease in 27 subjects following mRNA/DNA SARS-CoV-2 vaccination. Vaccines.

[71] Skroza N., Bernardini N., Tolino E., Proietti I., Mambrin A., Marchesiello A., Marraffa F., Rossi G., Volpe S., Potenza C. (2021). Safety and impact of anti-COVID-19 vaccines in psoriatic patients treated with biologics: A real life experience. J. Clin. Med..

[72] Damiani G., Allocco F., Malagoli P., Young Dermatologists Italian Network (2021). COVID-19 vaccination and patients with psoriasis under biologics: Real-life evidence on safety and effectiveness from Italian vaccinated healthcare workers. Clin. Exp. Dermatol..

[73] Megna M., Potestio L., Gallo L., Caiazzo G., Ruggiero A., Fabbrocini G. (2021). Reply to “Psoriasis exacerbation after COVID-19 vaccination: Report of 14 cases from a single centre” by Sotiriou, E.; et al. J. Eur. Acad. Dermatol. Venereol..

[74] Musumeci M.L., Caruso G., Trecarichi A.C., Micali G. (2021). Safety of SARS-CoV-2 vaccines in psoriatic patients treated with biologics: A real life experience. Dermatol. Ther..

[75] Talamonti M., Galluzzo M. (2021). Safety of COVID-19 vaccines in patients with psoriasis undergoing therapy with anti-interleukin agents. Expert Opin. Biol. Ther..

[76] Ramasamy M.N., Jessop L.J. (2021). CoronaVac: More data for regulators and policy makers. Lancet.

[77] Carugno A., Gambini D.M., Raponi F., Vezzoli P., Test E.R., Arosio M.E.G., Callegaro A., Sena P. (2020). Coronavirus disease 2019 (COVID-19) rash in a psoriatic patient treated with Secukinumab: Is there a role for interleukin 17?. Dermatol. Ther..

[78] Rosi E., Fastame M.T., Scandagli I., Di Cesare A., Ricceri F., Pimpinelli N., Prignano F. (2021). Insights into the pathogenesis of HS and therapeutical approaches. Biomedicines.

[79] Seltzer J.A., Okeke C.A., Perry J.D., Shipman W.D., Okoye G.A., Byrd A.S. (2020). Exploring the risk of severe COVID-19 infection in patients with hidradenitis suppurativa. J. Am. Acad. Dermatol..

[80] Adenote A., Dumic I., Madrid C., Barusya C., Nordstrom C.W., Prada L.R. (2021). NAFLD and infection, a nuanced relationship. Can. J. Gastroenterol. Hepatol..

[81] Marzano A.V., Moltrasio C., Genovese G., Muratori S., Dapavo P., Fabbrocini G., Patrizi A., Sechi A., Micali G., Pellegrino M. (2020). Hidradenitis suppurativa and adalimumab in the COVID-19 era. Eur. J. Dermatol..

[82] Molinelli E., Diotallevi F., Simonetti O., Brisigotti V., Sapigni C., Radi G., Campanati A., Offidani A. (2020). Management of patients with hidradenitis suppurativa during the COVID-19 pandemic: Risk and benefit of immunomodulatory therapy. Dermatol. Ther..

[83] Rozzo G., Ramondetta A., Fierro M.T., Dapavo P., Ribero S. (2020). Moderate-to-severe hidradenitis suppurativa under systemic therapy during the COVID-19 outbreak. Dermatol. Ther..

[84] Marasca C., Ruggiero A., Megna M., Annunziata M.C., Fabbrocini G. (2020). Biologics for patients affected by hidradenitis suppurativa in the COVID-19 era: Data from a referral center of Southern Italy. J. Dermatol. Treat..

[85] Galán J.L., Silvente C., González M., García C., Díez K., Martín M., Velázquez D., de la Cueva P. (2020). Experience in patients with hidradenitis suppurativa and COVID-19 symptoms. J. Am. Acad. Dermatol..

[86] Lima X., Cueva M., Alora M. (2020). COVID-19 in patients with hidradenitis suppurativa. Br. J. Dermatol..

[87] Giamarellos-Bourboulis E.J., Bettoli V., Jemec G.B.E., del Marmol V., Marzano A.V., Prens E.P., Tzellos T., Zouboulis C.C. (2021). Anti-COVID-19 measurements for hidradenitis suppurativa patients. Exp. Dermatol..

[88] Freeman E.E., McMahon D.E., Lipoff J.B., Rosenbach M., Kovarik C., Desai S.R., Harp J., Takeshita J., French L.E., Lim H.W. (2020). The spectrum of COVID-19–associated dermatologic manifestations: An international registry of 716 patients from 31 countries. J. Am. Acad. Dermatol..

[89] Montero-Vilchez T., Martinez-Lopez A., Salvador-Rodriguez L., Molina-Leyva A., Arias-Santiago S. (2020). Management of patients with hidradenitis suppurativa during the COVID-19 pandemic. Dermatol. Ther..

[90] Marasca C., Ruggiero A., Napolitano M., Fabbrocini G., Megna M. (2020). May COVID-19 outbreaks lead to a worsening of skin chronic inflammatory conditions?. Med. Hypotheses.

[91] Marasca C., Fabbrocini G., Barrea L., Capasso G., Di Guida A., Cinelli E., Fontanella G. (2021). Endocrinological disorders and inflammatory skin diseases during COVID-19 outbreak: A review of the literature. Minerva Endocrinol..

[92] Rosi E., Pimpinelli N., Prignano F. (2020). Is biologic treatment of hidradenitis suppurativa during the COVID-19 pandemic different from psoriasis biologic treatment?. J. Dermatol. Treat..

[93] Price K.N., Collier E.K., Grogan T.R., Hsiao J.L., Shi V.Y. (2021). Hidradenitis suppurativa patient perspectives during the COVID-19 pandemic. Dermatol. Online J..

[94] Caro R.D.C., Pensa C., Lambiase S., Candi E., Bianchi L. (2021). May COVID-19 infection induce a paradoxical improvement of a non-responsive case of hidradenitis suppurativa?. Ital. J. Dermatol. Venereol..

